# Thermal Imaging Following Exercise in Working Dogs

**DOI:** 10.3389/fvets.2021.705478

**Published:** 2021-09-06

**Authors:** Calan M. Farley, Patricia Kaynaroglu, Donna Magness, Ronald J. Riegel, Cynthia M. Otto

**Affiliations:** ^1^Penn Vet Working Dog Center, University of Pennsylvania School of Veterinary Medicine, Philadelphia, PA, United States; ^2^School of Biomedical Engineering Science and Health Systems, Drexel University, Philadelphia, PA, United States; ^3^Digatherm Inc., Ocala, FL, United States

**Keywords:** treadmill, rubble search, canine sports medicine, muscle, conditioning, thermal imaging

## Abstract

Disaster search dogs traverse diverse and unstable surfaces found in collapsed buildings. It is unknown if the physical conditioning on a treadmill involves the same muscle groups that are involved in rubble search. This 14-week prospective cohort study was conducted to investigate changes within the thermal gradients of specific dog muscles following treadmill compared to rubble search. Nine dogs, ranging in age from 6 months to 4 years, were randomly assigned to one of two groups. Each week the two groups would participate in either 20 min of treadmill or rubble searches. Prior to exercise, the dogs were weighed and then kenneled in a temperature-controlled study room for 20 min at 21°C. Pre-exercise thermal images were then captured of the standing dog from the dorsal, left and right lateral, and caudal perspectives, and of the sitting dog from the rostral perspective. Following a 10-min warm-up period of stretches, dogs proceeded to either treadmill or search. Upon completion, dogs were kenneled in the study room for 20 min prior to post-exercise thermal images. Images were sectioned into 22 muscle regions, the pre-exercise images were subtracted from the post-exercise images to determine the temperature difference (ΔT) for that dog, on that day, for that activity. Thermography measures radiant energy, temperature, and converts this information into an image. This study looked at ΔT within a region pre and post-exercise. The study failed to find a statistically significant difference in the ΔT within each muscle group between treadmill and search activities. There was a decrease in ΔT within all muscle regions over the of the study except for the right cranial shoulder, right caudal shoulder, and right hamstring for the treadmill activity only. The decrease was significant in the pelvis, left longissimus, right cranial shoulder for the search activity, left oblique, left caudal shoulder, and left quadricep muscular regions. These findings suggest that ΔT in muscle groups are similar between treadmill exercise and rubble search. Regardless of the exercise type, 14 weeks of structured Search and Rescue training and treadmill exercise resulted in less ΔT associated with a structured weekly exercise.

## Introduction

During periods of activity, as a byproduct of contracting and performing work, muscles produce heat. In a study by González-Alonso et al., investigating the production of heat in human muscles during exercise, it was found that heat production by muscles doubled over a period of 3 min while subjects maintained a constant power output (1). This heat produced by the muscles will either be stored within the muscle tissue, removed to the body core via circulation, or transferred to the skin via conduction and convection (1, 2). Infrared Thermography (IRT) involves the use of instrumentation to detect the thermal energy being emitted from the skin. The levels of energy being emitted directly correlates to the circulation within the muscles underlying the skin, and the detection of both increasing (increased temperature, hyperthermia) and decreasing (decreased temperature, hypothermia) blood flow (3, 4). This technology has become more readily accessible for consumer use and is capable of being employed as a tool in a variety of fields. Such uses include identification of local areas of hyperthermia (increased circulation), which correlates to possible injury, as demonstrated in a study of racing greyhounds evaluating preferential limb utilization during racing (5). Other applications include the evaluation of dog biomechanics and gait analysis (6), identification of musculoskeletal disorders such as hip osteoarthritis in dogs (7, 8), and detection of tendonitis in racehorses (9). Thermography has also been used to investigate skin temperature changes after exercise on a treadmill with findings that skin temperature increases with a correlation to muscle activity (10, 11). A study by Repac et al., evaluating muscle activity in dogs after 6 min of walking on a treadmill, found a significant increase in surface temperature, between pre and post-exercise, correlating to biceps femoris and gracilis muscle activity (12).

Disaster search dogs are a subset of working dogs that are trained and employed in disaster zones to locate missing persons. These dogs undergo extensive physical conditioning and training to traverse various obstacles to ensure that they are physically fit and capable of navigating over the varied obstacles and difficult terrain found in a disaster rubble piles. The Penn Vet Working Dog Center trains dogs for a variety of careers including disaster search and rescue, and a key component of those dogs training comes from trotting on a treadmill. While the treadmill allows for physical conditioning, it is unknown if trotting on a treadmill activates muscles used in locomotion in a comparable pattern and extent as during rubble search which would make treadmill a valuable addition to Search and Rescue dogs training regime.

Infrared thermal imaging technology offers a method to indirectly investigate differential dog muscle activity after exercise on a simulated disaster rubble pile compared to treadmill exercise (10–12). It is hypothesized that the circulation, visualized by infrared thermal imagery, of dog muscles following treadmill exercise would be different than that following rubble search, due to the different movement patterns required for the two activities. Additionally, it was theorized that a regular structured weekly exercise regime would lead to decreased temperature difference within the musculature over the course of the study regardless of the type of exercise. This change in circulation (temperature difference; ΔT) can be determined by obtaining thermal images pre and post-exercise, and then calculating the difference in temperature (ΔT).

## Methods

### Animals

In this University of Pennsylvania Institutional Animal Care and Use Committee (IACUC protocol #806076) approved study, 3 German Shepherds, and 6 Labradors participated. Two of the Shepherds and one Labrador were female, the rest were male, four of the males were neutered. Two of the Labradors did not participate for the full study period, one was present for the first 6 weeks of the study, while the other was present for 5 weeks of the study. The data gathered from the dogs that did not participate in the full study was incorporated in the data analysis. Male dogs weighed 26.4 ± 2.59 kg, female dogs weighed 23.9 ± 2.18 kg. One dog was 6 months old; one dog was 9 months old; 4 dogs were between 15 and 18 months old, and 3 dogs were between 2 and 4 years old at the start of the study. Throughout the study, all dogs remained in good physical health based on veterinary examination. To participate in the study, all dogs were required to be capable of a continuous trot on the treadmill for 20 min and to conduct 20 min of searching for humans on a rubble pile. All of the dogs in the study were in training at the Penn Vet Working Dog Center. The training program is focused on olfactory detection with a secondary focus of improving dog health and wellness. The dogs are engaged in training 5 days a week, the type and amount of training varies based upon environmental conditions and staff and volunteer availability. The physical training includes obedience, search and rescue training, stretching and core muscle exercises, directional control at a distance, treadmill, and obstacle traversal training for periods of 20–30 min at a time.

### Study Design

The study lasted for a period of 14 weeks. The first week served as a pilot study to allow for familiarization with the thermal imaging system and the study protocol, the remaining 13 weeks were utilized to gather data with 1 of those 13 weeks being used as a makeup week for any dogs that failed to participate in one of the previous weeks of data collection. In this crossover design, the dogs were randomly divided into two groups, with one group participating in the study on the Thursday of each week, while the other group participated on the Friday of each week. Each week the two groups would either do treadmill or rubble searches as their activity for that week. All dogs in the same group would perform the same activity that was assigned for that week. The activity that one group performed did not affect what activity the other group would perform that week. It was randomly determined what activity each group would conduct for each week of the study.

### Timing

On all study days with treadmill activity, the dog handler was responsible for timing. After 20 min based on the treadmill timer, the handler would stop the treadmill, terminating the activity. During search activity, there was a separate timer. The timer was responsible for marking the initiation and termination of the search activity. The timer was responsible for providing 10-, 5-, 2-, and 1-min warnings to the handler to ensure that the search activity was terminated after 20-min of searching had occurred.

### Exercise Protocol

**Figure 1:**
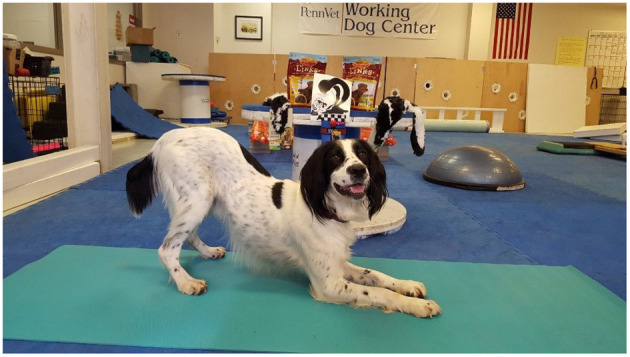
Play bow stretch.

**Figure 2:**
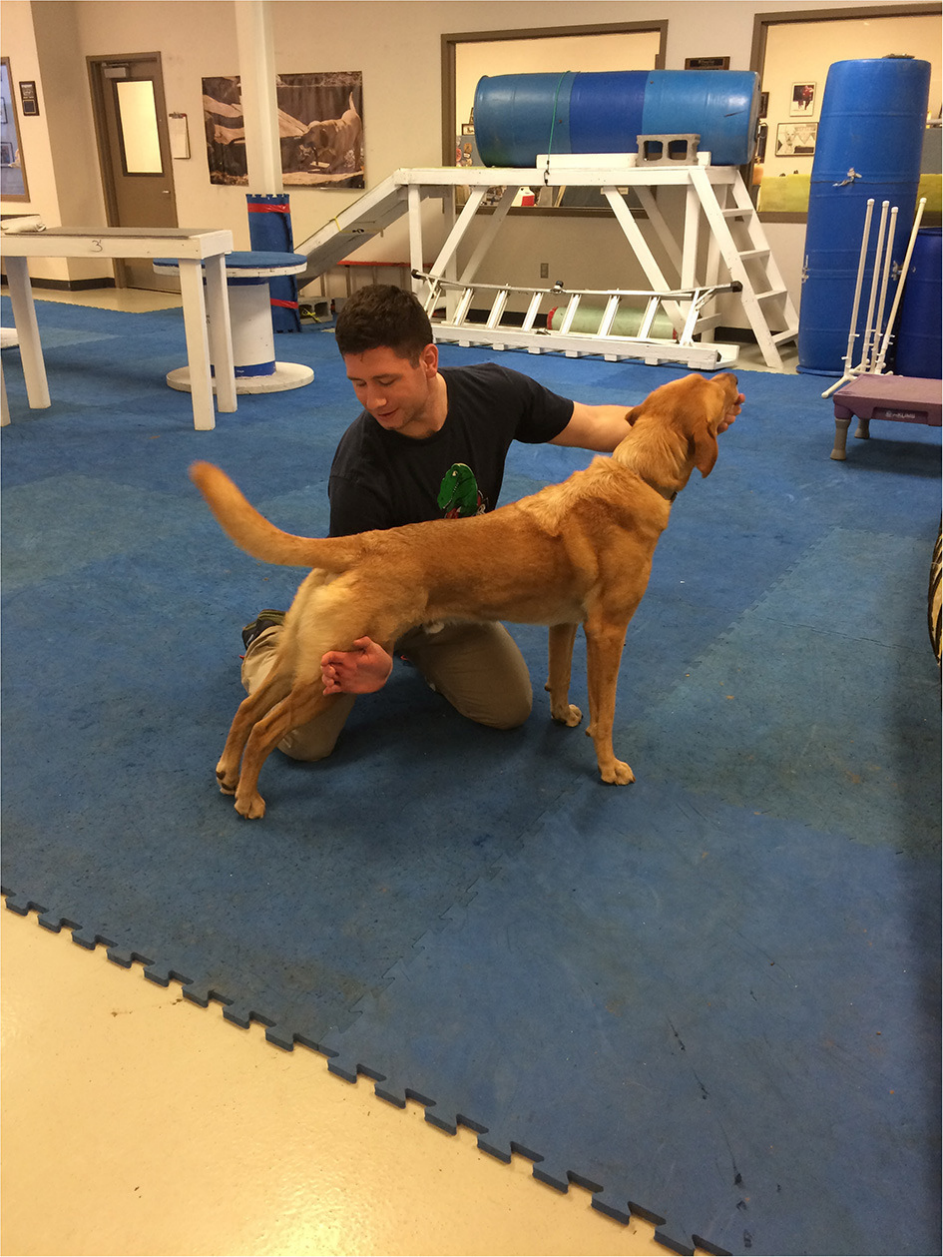
Counter stretch.

**Figure 3:**
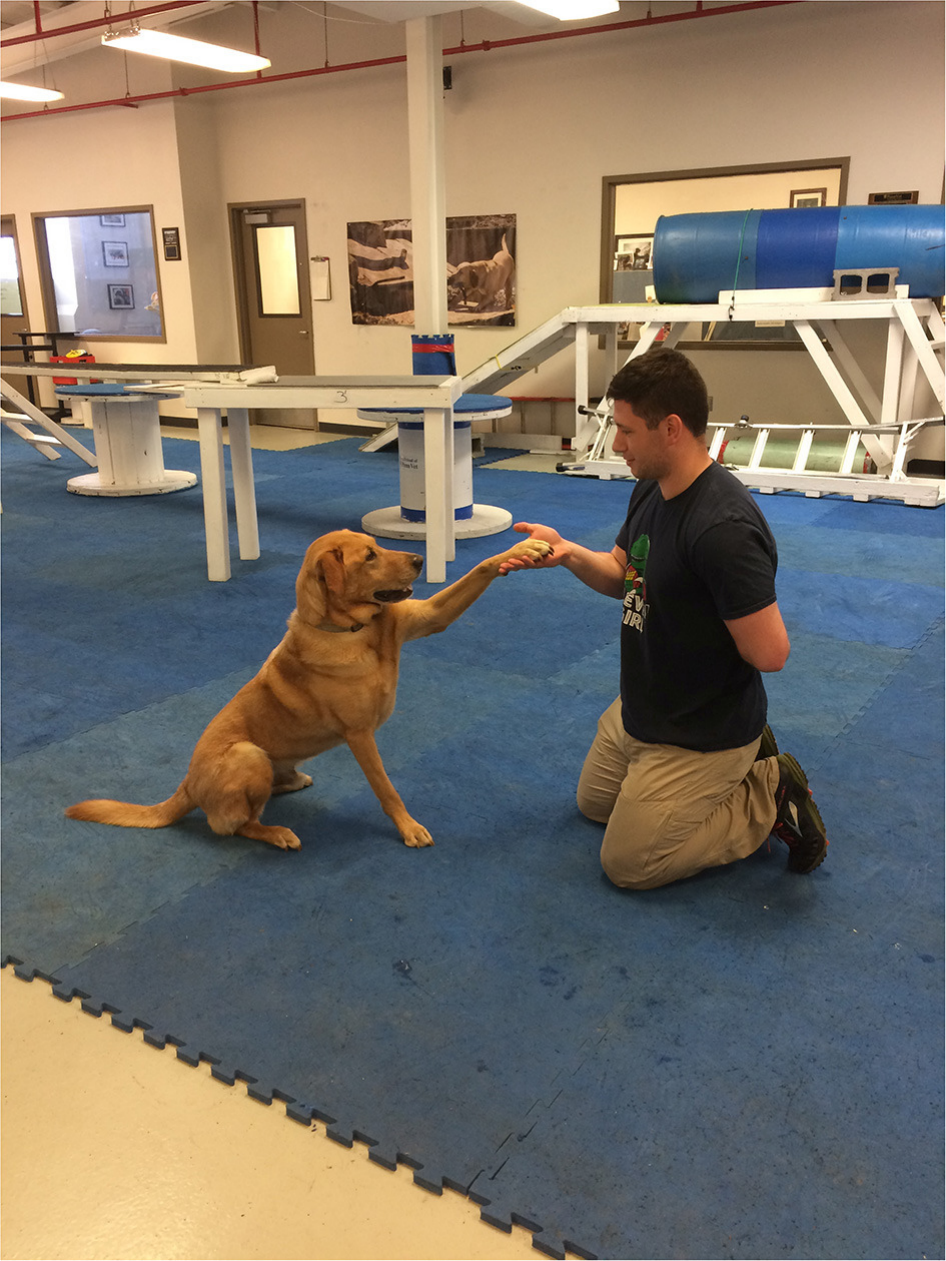
High five stretch.

**Figure 4:**
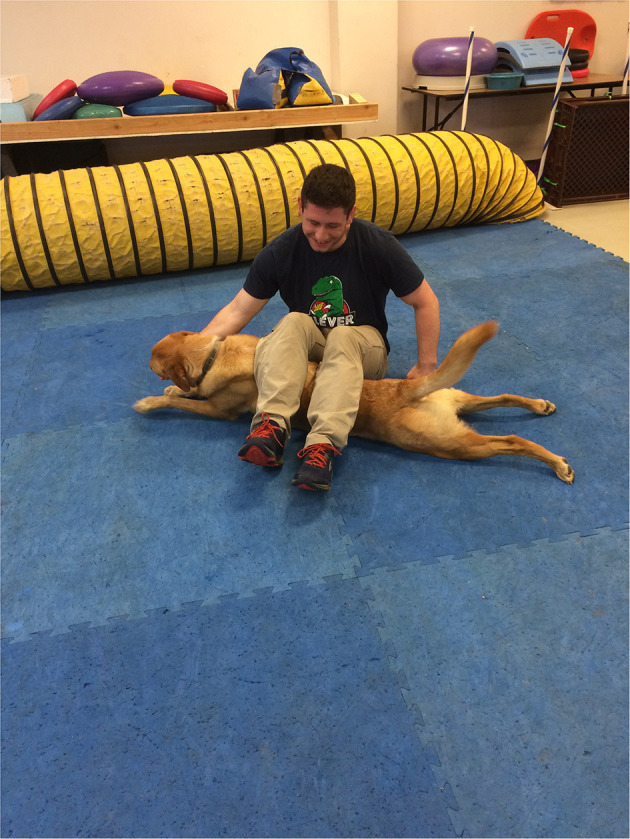
Frog stretch.

**Figure 5:**
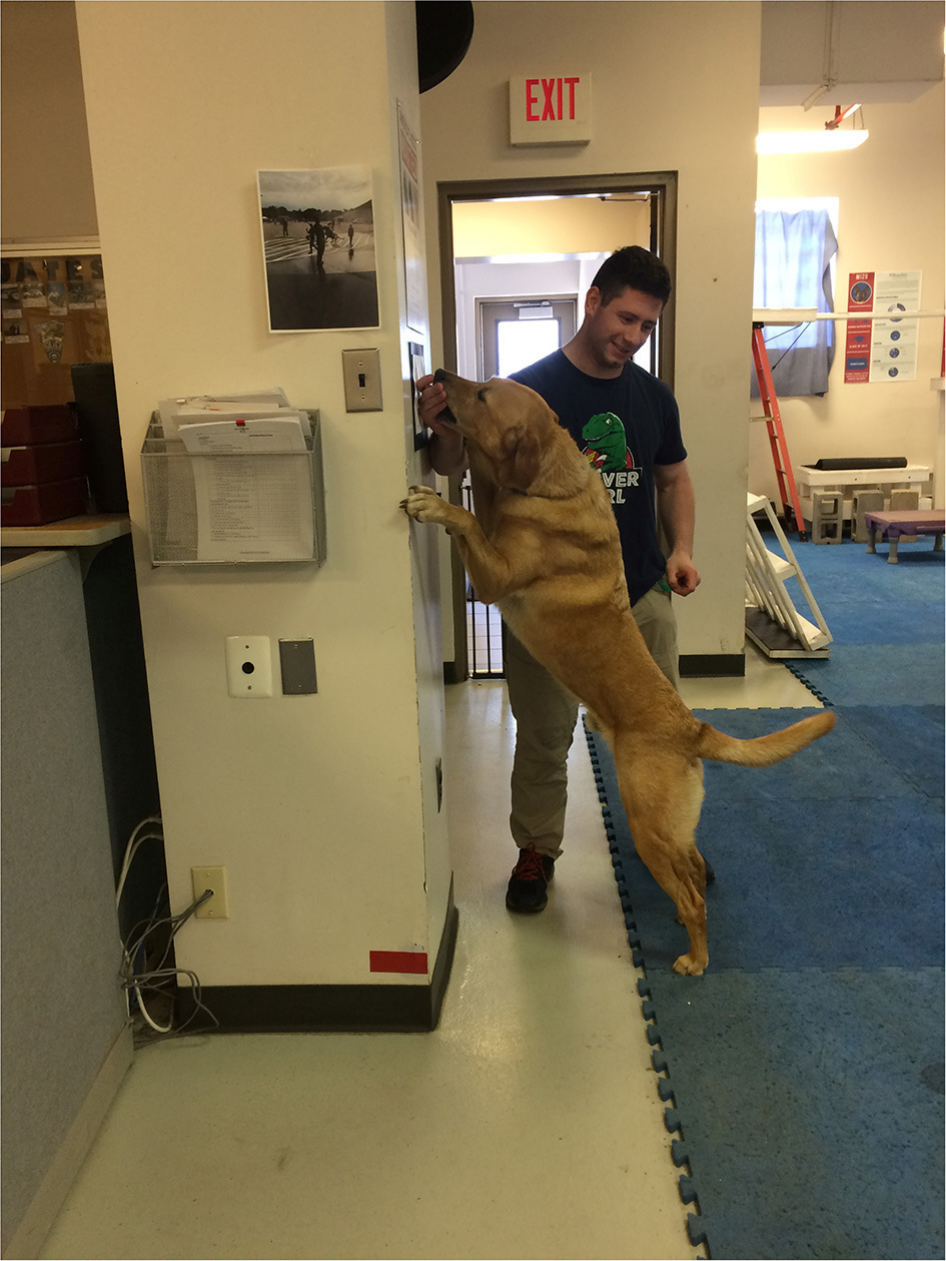
Paws up stretch.

**Figure 6:**
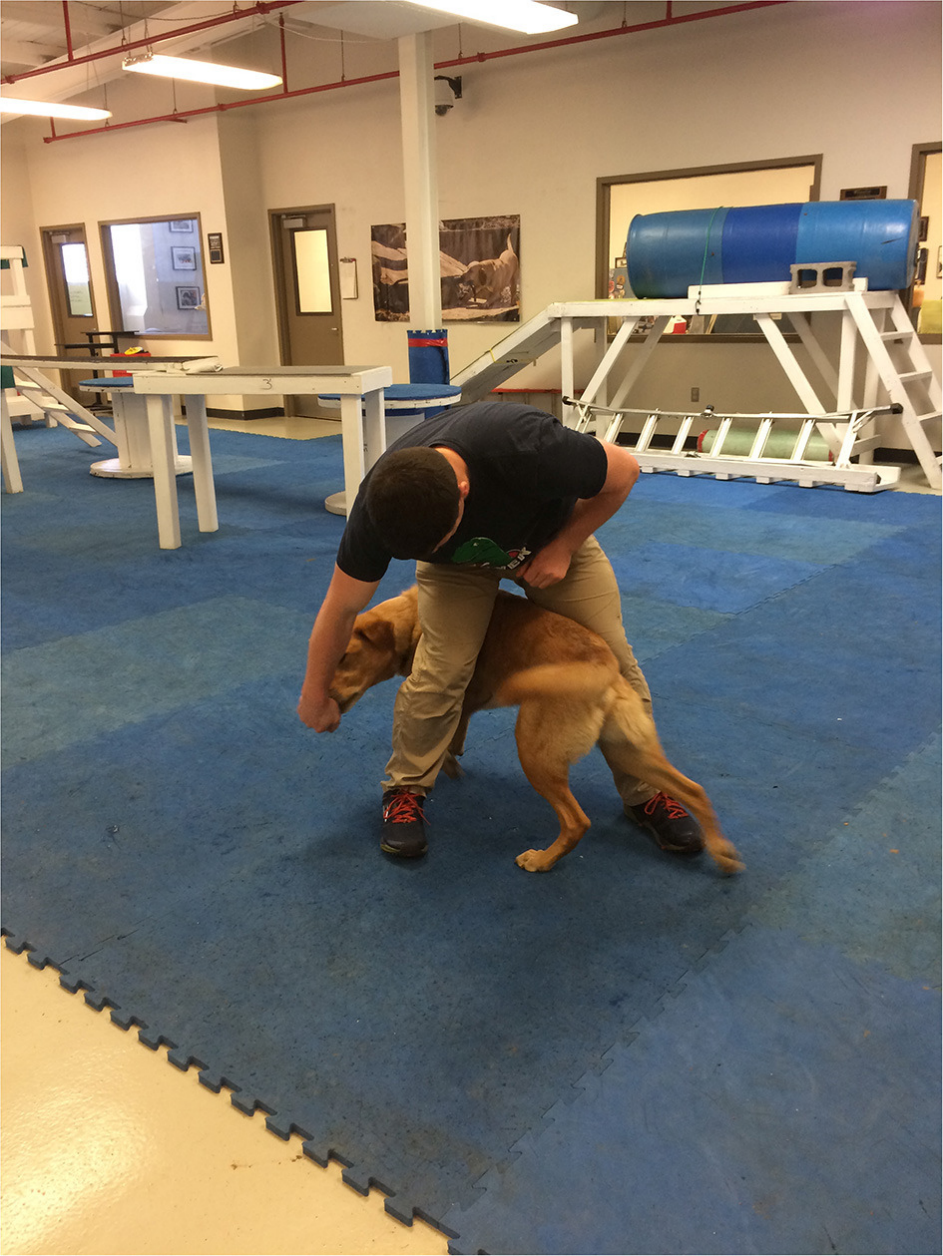
Figure eight stretch.

The study based its thermographic imaging protocol upon the imaging protocol established in a previous study (13). At the beginning of each study day, the dogs were individually allowed to equilibrate in a 21°C temperature-controlled room, where the dog body weight was obtained (13). The dogs were allowed to assume a position of their choosing to minimize any stress. The dog handler wore gloves to minimize any heat transfer due to accidental contact between the handler and the dog and prevent dog fur displacement. The gloves utilized were cold weather gloves, the handler would put on the gloves immediately prior to handling the dog during the imaging sessions and would take off the gloves upon the completion of the thermal imaging. While positioning the dog for thermal imaging capture, the handler would only touch the dog on their head and tail to avoid interfering with the areas the study was attempting to capture (13). After the thermal images were captured, all dogs completed a warm-up exercise period of stretching that lasted 10 min, and consisted of 5 repetitions of play bows, counter stretching, high fives, frogs, paws up, and figure eights, each repetition lasting 5–10 s. In the play bow stretch, the dog lowers their head and front legs, while remaining standing on their rear legs and stretches their abdominal and hindlimb muscles (Figure 1). In the counter stretch, the dog leans forward and rolls onto the toes of the hindlimbs, stretching their hindlimb muscles (Figure 2). During the high five stretch, the dog is in a sitting position and places their front paw into the handler's hand, the handler than slowly raises the dog's paw and front limb upwards, stretching the front leg and shoulder muscles, each repetition includes both front limbs (Figure 3). During the frog stretch, the handler sits on the ground with the bottom of their feet touching the ground, the handler's legs form an inverted “v,” the dog crawls through the gap under the handler's legs, stretching the pelvic muscles and extending the hind limbs behind them (Figure 4). During the paws up stretch, the dogs stand upright on their hindlegs and place their front legs against a wall, this stretches the muscles of the rear leg (Figure 5). In the figure eight stretch, the handler stands with their feet shoulder width apart, the dog then weaves between the handler's legs stretching their lateral abdominal muscles (Figure 6). Upon completion of the stretches the dog performed either the treadmill or rubble search activity for 20 min. After the activity period, the dog was returned to the study room, offered water and placed into the kennel. After 20 min, thermal images were taken of the dog to determine post-activity muscle temperature.

**Figure 7:**
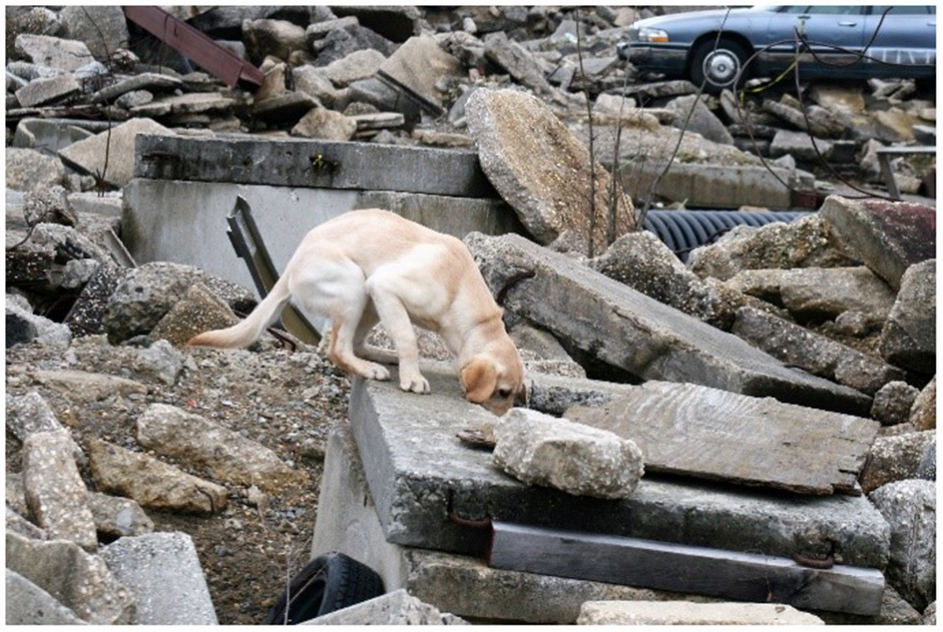
Dog searching on rubble pile.

A level (0 degree incline) treadmill with a belt length of 77 inches, a belt width of 17 inches and a maximum belt speed of 7 mph was used (Dogtread, Petzen Products, Ogden UT, https://dogtread.com/treadmills/). During treadmill exercise, the belt speed was adjusted to maintain a continuous trot, with a speed range between 2 and 4 mph depending on the dog. If a dog left the treadmill, the treadmill would be restarted, and the dog would continue trotting for the remaining time. During the treadmill exercise, the dog is maintaining a relatively constant speed and traveling on a flat surface. For the search activity, the dogs would perform rubble searches, upon locating the hidden person, a game of tug was initiated as a reward before resuming searching for the next victim. The dogs would find 3–4 victims on average over the course of the 20 min of rubble searching. The rubble pile on which the dogs searched consisted of uneven terrain made up of concrete slabs, wooden slats, and vehicles (Figure 7). During rubble activity, the dog's speed is variable, and the dog is required to climb over various obstacles this could potentially lead to different patterns and intensity of muscle activity when compared to the treadmill exercise. To prevent overheating during rubble searches, dogs were offered water if they displayed signs of overheating such as panting, shade seeking, eye squinting, or if their tongue became flattened and curled upwards (14).

### Data Collection

A digital thermal imaging device (Digatherm IR Tablet 640, Digatherm by Infrared Cameras Inc., Beaumont, TX, http://digatherm.com/) was used to obtain images of the standing dog from the dorsal, left and right lateral, rostral, and caudal perspectives. Additionally, a thermal image was taken from the ventral perspective when the dog was in a sitting position. The camera has a thermal sensitivity of <0.02°C at 30°C and an accuracy of ±1°C. For analysis purposes, the dorsal, both lateral, and sitting ventral perspectives were used to thermally analyze the different peripheral dog muscle regions. The thermal imaging device had software that allowed specific regions to be created in a captured image. This software provides the minimum, maximum, standard deviation, and mean temperatures inside the selected region.

**Figure 8:**
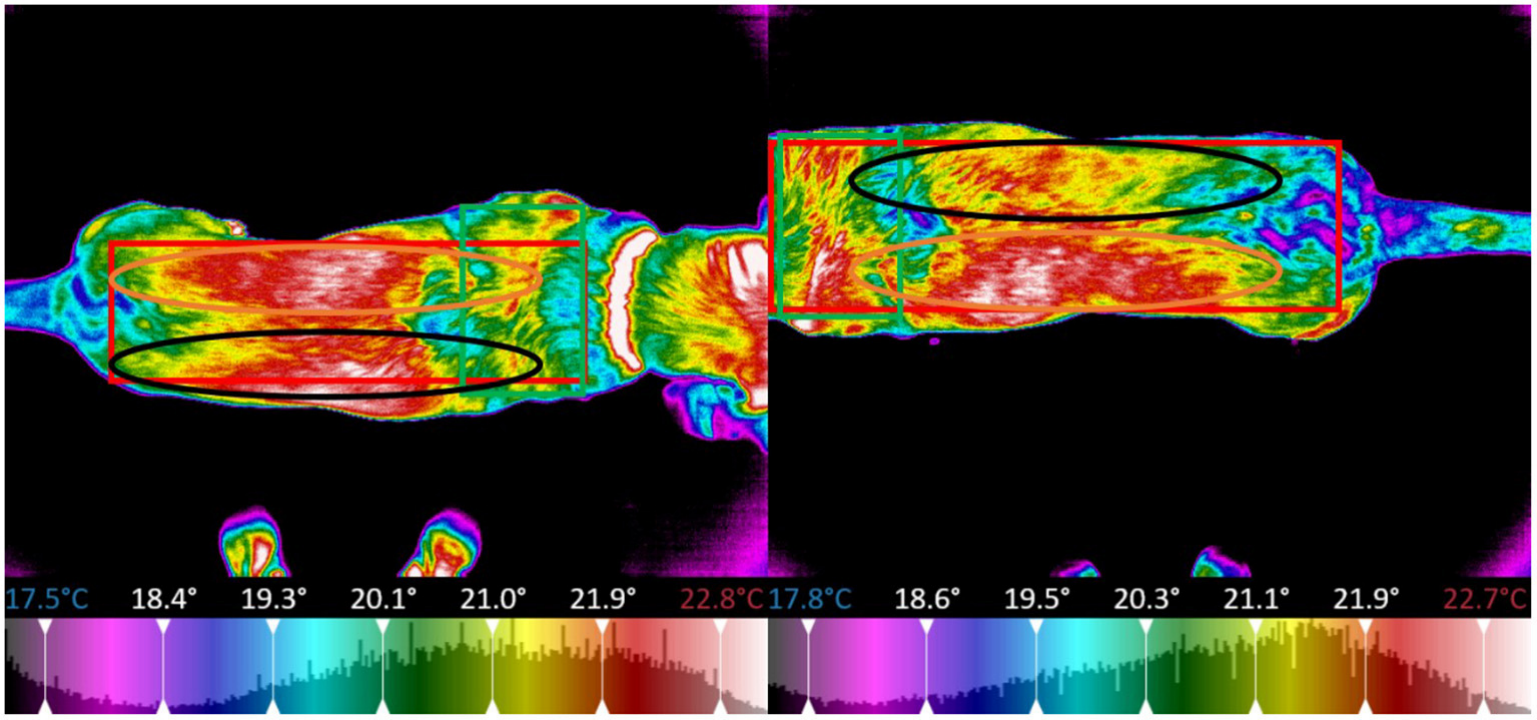
Sectioned muscle regions dorsal view (Left, pre-exercise; Right, post-exercise).

**Figure 9:**
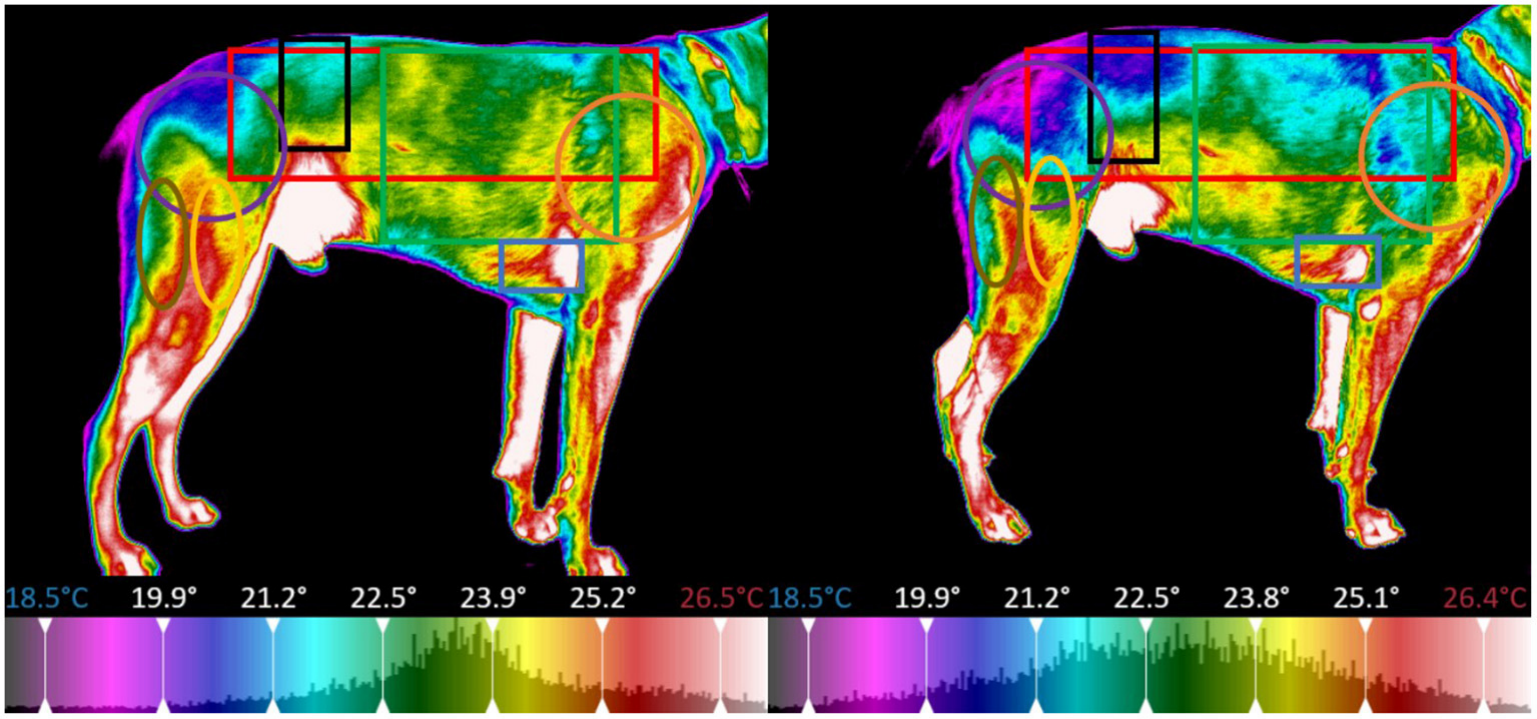
Sectioned muscle regions right lateral view (Left, pre-exercise; Right, post-exercise).

**Figure 10:**
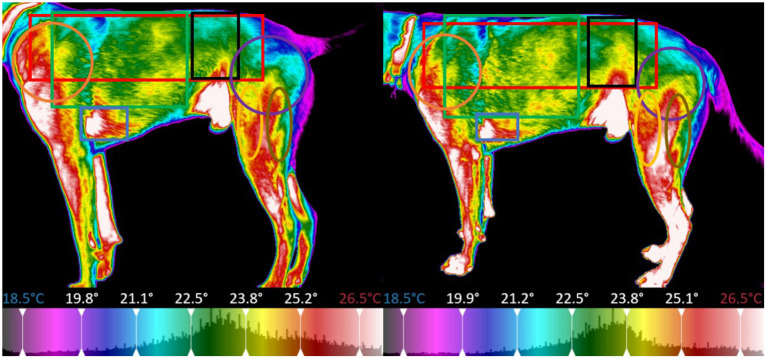
Sectioned muscle regions left lateral view (Left, pre-exercise; Right, post-exercise).

**Figure 11:**
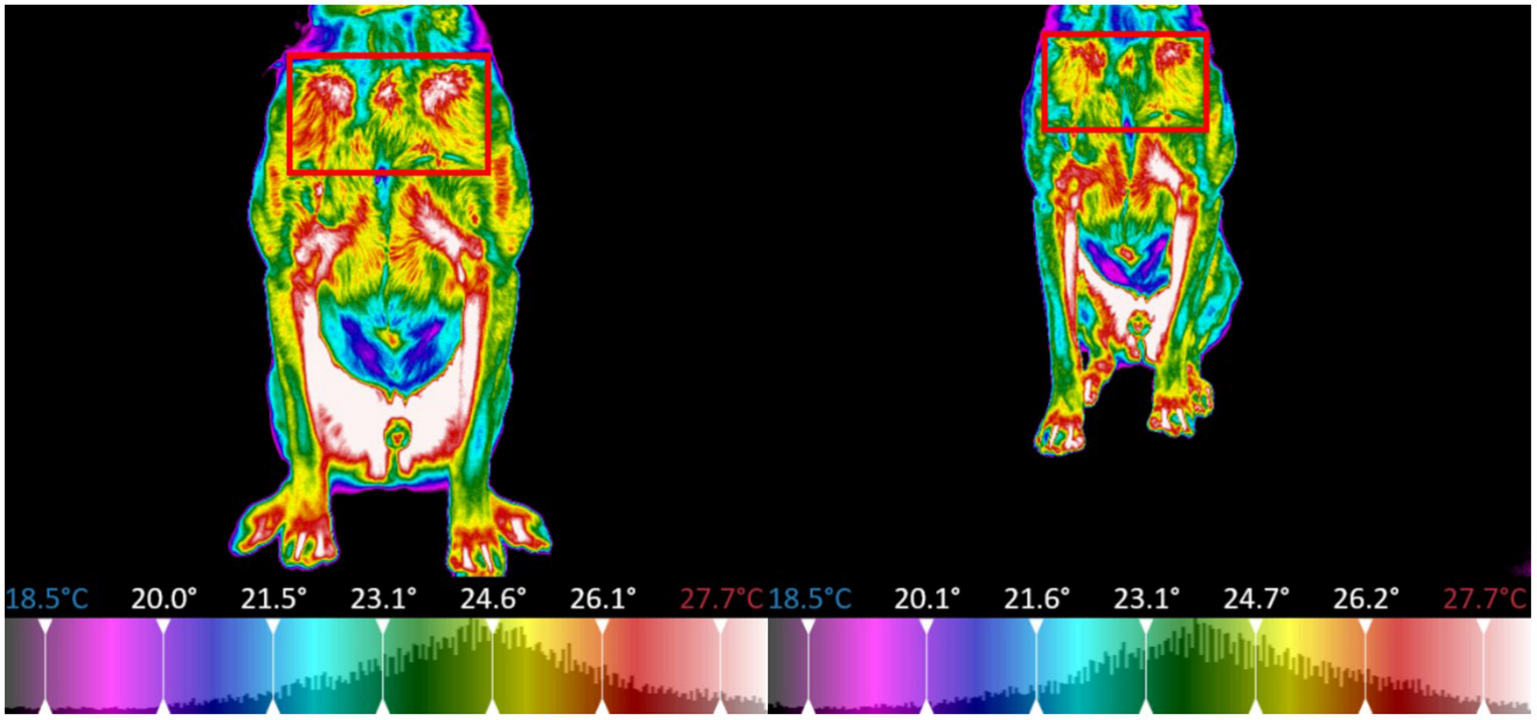
Sectioned muscle regions ventral view (Left, pre-exercise; Right, post-exercise).

The thermal images of the dog were segmented into regions centered around specific major muscle groups and the minimum, maximum, mean, and standard deviation of temperature in degrees Celsius for each segment was calculated. For all thermal images the dogs were placed on top of a 5-inch platform which was set in a fixed position with a white brick wall providing a uniform background per the protocol established by Loughin and Marino (13). The platform was positioned so that it was not directly located under any lights to prevent any radiant heat from affecting the thermal image capture. All images were taken at a distance of 0.3 meters away from the dog. The dogs were on leash and led onto the platform for thermal image capture, to reposition for other perspectives, the dogs were led off the platform and directed to turn and then led back onto the platform. The thermal imager automatically adjusted its temperature scale to reflect the temperature range of the room and dog in the image, the operator of the imager ensured that the temperature scale of the pre-exercise image matched that of the post-exercise image for each dog, allowing the temperature difference to be calculated for each dog on each study day. Because this study was focused on temperature difference pre and post-exercise, each dog served as their own control (i.e., the temperature range for all the images were the same for the pre and post-exercise image for a specific dog on a specific day), the temperature range could vary between dogs and between study days. The temperature range was adjusted to place the entire color palette on the dog. This eliminated any temperature readings from the background. From the dorsal perspective imagery, the dog was segmented into a global region, as well as sub-regions centered on the trapezius, left and right longissimus, and pelvic area (Figure 8). For the left and right lateral perspective imagery, the dog was segmented into a global region, as well as smaller sub-regions centered around the latissimus dorsi, pectoralis, obliques, front shoulder, rear shoulder, hamstring, and quadricep regions (Figures 9, 10). The ventral perspective was segmented into chest region (Figure 11). For each dog on each day ΔT was calculated as the average temperature of each muscle region post-activity minus the average temperature of each muscle region pre-activity.

### Statistical Analysis

The thermal images and data were inspected by a trained technician to ensure that there were no software errors in the obtained imagery. A linear regression was performed for both search and treadmill data to determine the ΔT trend over the course of the study. The linear regression was conducted separately on both the search and the treadmill activities across all muscle groups. Polynomial curve fitting was employed to determine the best fitting linear equation for each activity and muscle group over the course of the entire study. An Analysis of Variance (ANOVA) was conducted using the baseline (week 2) search and treadmill data across all dogs for each muscle region, to see if there was a significant difference in the ΔT between search and treadmill activities, or if there was a significant difference in dog ΔT within each activity, there was 9 dogs used in this ANOVA. A repeated measure ANOVA was run to determine if there was a significant change in average ΔT in either the search or treadmill activity over the course of the entire study. The repeated measure ANOVA looked at the average ΔT of all dogs for each muscle group and activity across every week of the study. The number of dogs used in the ANOVA decreased from 9 to 8 after week 5, and from 8 to 7 after week 6 of the study; the repeated measure ANOVA was chosen because it allows for a change in the number of dogs without that change invalidating the ANOVA's findings. For this study an alpha value of 0.05 was used.

## Results

All dogs, except for the two that left the study early, conducted 6–7 rubble searches and 5–6 treadmill sessions over the course of the study depending on which group they were assigned to. Dogs in group A conducted 6 search sessions and 6 treadmill sessions, dogs in group B conducted 7 search sessions and 5 treadmill sessions.

**Figure 12:**
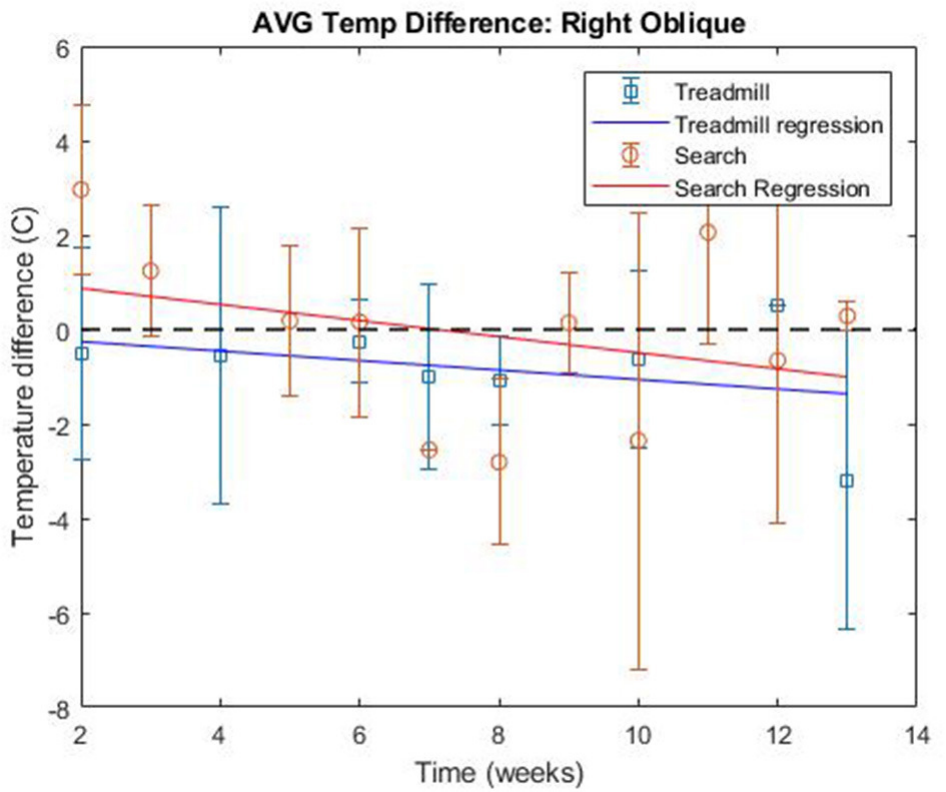
Average thermographic intensity difference over study time for right oblique region.

The linear regression of the search data over the course of the study, found a trend of decreasing ΔT over study time for all muscle regions. The average slope of the regression across all muscle groups was −0.2560, with a standard deviation of 0.0802 between the muscle groups (Table 1 and Figure 12).

**Table 1:** Regression slope data for treadmill and search data across all muscle groups.

**Regression slope**		
**Number of dogs = 9**	**Treadmill**	**Search**
Average	−0.1316	−0.2560
Standard deviation	0.0816	0.0802
Back global	−0.1561	−0.3227
Trapezius	−0.2140	−0.2656
Pelvis	−0.1385	−0.3232
Right longissimus	−0.1689	−0.3257
Left longissimus	−0.2097	−0.3619
Right lateral global	−0.0494	−0.1830
Right latissimus	−0.1390	−0.2184
Right pectoralis	−0.0610	−0.2867
Right oblique	−0.1005	−0.1705
Right cranial shoulder	0.0461	−0.0703
Right caudal shoulder	−0.0122	−0.2003
Right quadricep	−0.1009	−0.2816
Right hamstring	−0.0010	−0.1648
Left lateral global	−0.1845	−0.2051
Left latissimus	−0.1667	−0.2188
Left pectoralis	−0.2133	−0.3035
Left oblique	−0.2283	−0.2515
Left cranial shoulder	−0.0640	−0.1466
Left caudal shoulder	−0.1944	−0.2982
Left quadricep	−0.2244	−0.3937
Left hamstring	−0.2265	−0.3445
Chest	−0.0874	−0.2945

**Figure 13:**
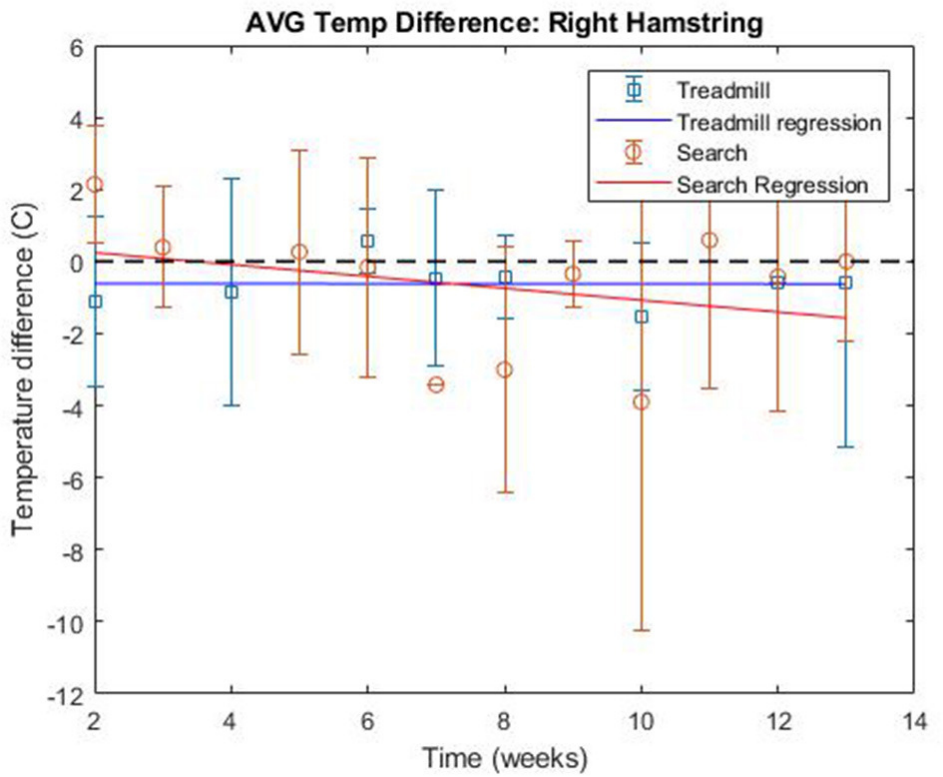
Average thermographic intensity difference over study for right hamstring region.

The linear regression of treadmill data over the course of the study, found a trend of decreasing ΔT over study time for all muscle regions except for the right cranial shoulder, the right caudal shoulder, and the right hamstring. The average slope of the regression across all muscle groups was −0.1316, with a standard deviation of 0.0816 between the muscle groups (Table 1 and Figure 13). The right cranial shoulder, the right caudal shoulder, and right hamstring saw negligible changes in ΔT over the course of the study with regression slopes of 0.0461, −0.0122, and −0.0010, respectively.

Using the two-way ANOVA for the week 2 search and treadmill data, no detectable difference was found between the ΔT during search vs. treadmill for any muscle region (Table 2). The trapezius displayed a statistically significant variation of ΔT among all the dogs participating in the treadmill exercise during week 2 of the study with a *p*-value of 0.0411 (Table 2).

**Table 2:** ANOVA testing for week 2 treadmill and search data, and for all data over study time.

**Number of dogs = 9**	**Activity ANOVA**	**Activity ANOVA**	**Time ANOVA**
**Region**	**Between activity** ***p*** **-value**	**Within activity** ***p*** **-value**	**Time** ***p*** **-value**
Dorsal global	0.1163	0.0823	0.0662
Trapezius	0.2364	0.0411	0.0994
Pelvis	0.1105	0.1087	0.0059
Right longissimus	0.1963	0.1882	0.2619
Left longissimus	0.1533	0.0752	0.0243
Right lateral global	0.1546	0.5295	0.3873
Right latissimus	0.2042	0.5248	0.3676
Right pectoralis	0.2941	0.9579	0.5181
Right oblique	0.0986	0.4033	0.2903
Right cranial shoulder	0.1213	0.6014	0.0442
Right caudal shoulder	0.1183	0.7117	0.4571
Right quadricep	0.1341	0.5518	0.3846
Right hamstring	0.2192	0.8709	0.7905
Left lateral global	0.1181	0.0631	0.0777
Left latissimus	0.1632	0.0633	0.1032
Left pectoralis	0.4261	0.4320	0.1181
Left oblique	0.2226	0.1767	0.0324
Left cranial shoulder	0.0682	0.0530	0.2134
Left caudal shoulder	0.2207	0.2877	0.0412
Left quadricep	0.1662	0.4804	0.03
Left hamstring	0.1905	0.3757	0.0697
Chest	0.1716	0.5998	0.4273

*Mean and standard deviation of temperature difference over study time for search and treadmill. Green values are significant*.

The pelvis, the left longissimus, the left oblique, the left caudal shoulder, and the left quadricep muscular regions all showed a significant decrease in ΔT over the course of the study, while the right cranial shoulder showed a significant decrease in ΔT over the course of the study for the search activity only (Table 2).

## Discussion

No significant difference in ΔT could be detected between the search and treadmill activities suggesting that exercise on the treadmill activity leads to similar detectable thermal gradient changes implying similar patterns of muscle activity between treadmill and rubble search.

Over the course of the study for the search activity all muscle regions showed a trend of decreasing ΔT. Over the course of the study for the treadmill activity all muscle regions showed a trend of decreasing ΔT except for the right cranial shoulder, right caudal shoulder, and right hamstring. All the dogs involved in the study have been trained to heel on the handler's left side, and to frequently look at the handler to be alert for non-verbal commands. A study by Keebaugh et al. found that dogs will preferentially distribute their forelimb weight to the opposite side that the leash is on (15). Additionally, in a study conducted on the muscle temperature of racing greyhounds, it was determined that the greyhounds which were not leash trained, did not preferentially distribute their weight during locomotion (5). These studies suggest that leashing training could impact the results of the study. One potential explanation is that the dogs' preferential weight distribution during heeling on leash may have impacted the laterality of the results, however this study was not designed to evaluate dogs with leash training vs. those without and none of the study exercises were conducted on leash.

The results for the ANOVA evaluating ΔT over the course of the study found only 6 regions with a significant decrease in temperature difference. These regions were the pelvis, the left longissimus, and the right cranial shoulder for the search activity only, the left oblique, the left caudal shoulder, and the left quadricep. This trend of decreasing temperature suggests that the dogs' bodies became conditioned to rubble search and treadmill exercise over the course of the study. Search and rescue dogs undergo some degree of conditioning in response to the physical demands of their training regime. This conditioning effect results in several physiological changes in the dog specific to the activity that the dog is performing, any muscle or tissue that is not stressed by the activity will not become conditioned (16, 17). Conditioning can result in adaptations to the cardiovascular, skeletal, and muscular systems within the dog. Cardiovascular conditioning results in hypertrophy, or enlargement of the heart muscles and increased blood volume which increases the efficiency of transport of gases throughout the body and increases cardiac output resulting in better dog capacity to undergo strenuous physical activity (16, 17). Skeletal adaptations consist of increasing bone density and the enlargement of connective tissues allowing the dog body to better withstand stress. Muscular adaptations result in enlargement and change in the fiber composition of the muscles performing work (16, 17). Overall, the effect of conditioning results in the dog being more able to perform the specific activity for which it has been conditioned, for a longer duration without suffering from exhaustion or becoming overworked. For the treadmill and during periods of time while conducting rubble searches, the dog moved at a trot. The trot is an efficient, balanced gait in which diagonal limbs (i.e., left front and right rear), move forward and strike the ground at the same time (17). This form of locomotion is beneficial to the balanced conditioning of the dog as the dog's weight is born by one front and rear limb at a time, allowing for equal engagement of both sides of the body (17). All dogs, except for the two that left the study early, conducted 6–7 rubble searches and 4–5 treadmill searches over the course of the study depending on which group they were assigned to, in addition to their regular training at the Penn Vet Working Dog Center. The cumulative effect of the study activity sessions and their normal training regime outside of the study, potentially resulted in their muscles becoming conditioned to the treadmill and search activities as the study progressed, leading to decreased ΔT as muscles were more capable of performing the exercise and thus saw less muscle activity and heat over subsequent study weeks while performing those activities, the muscles that saw significant decreases in ΔT are all muscle groups located in the shoulders and legs and were involved in dog locomotion.

Statistically significant (*p* = 0.04) variation within the trapezius for the treadmill activity (Table 2) was seen in week 2. Given the small sample size, sampling bias could have an unduly large affect. Only 9 dogs were involved in the study, although not observed in this study, if one dog had an area with an increased thermal gradient resulting from lack of stretching or muscle injury during that study day it could have caused the statistically significant variation that was observed.

This study was limited by a small sample size due to the available population of dogs at the Penn Vet Working Dog Center capable of conducting 20 min of continuous trotting on the treadmill, or 20 min of searches on rubble. This limited sample size results in individual dog variation in ΔT having a much greater effect on the average ΔT for both the search and treadmill activities. Due to this small sample size, more research with a larger sample size is necessary to confirm and further investigate this observed conditioning effect.

The ages of the dogs ranged from 6 months to 4 years, although no formal studies have evaluated the effect of age on a dog's thermal gradients following exercise, in a study investigating thermal symmetry of skin temperature in human archers between 16 and 50 years of age, no detectable effect of age was found (18). The weekly study activity was randomized to prevent the study participants from being conditioned to a certain pattern of activity. The participants were also involved in regular physical training outside of study days, as such the study cannot determine whether the decreased ΔT over the course of the study was due to dog exercise in the treadmill activity, the search activity, or due to a combination of both activities, and cannot isolate the study activities from dog activity outside of the study.

The study took place in the fall/winter, there were no drastic changes of temperature during the study, the mean temperature during searches over the course of the study was 14.4°C, with a standard deviation of 6.4°C. Labradors and German Shepherds have different fur lengths and none of the dog's hair was clipped. A study by Loughin and Marino found that while there will be a difference in the mean temperature of clipped vs. unclipped dogs, the thermographic patterns remain similar (13). As this study was looking at the temperature difference between pre and post-activity, each dog served as their own control minimizing the effect that differing fur length would have on the study results. Another study by Vainionpää et al. on the usability of thermal imaging in dogs, found that different fur length caused no significant difference in the thermographic images obtained between 26 different breeds of dogs (19).

Dogs were kenneled for at least 20 min to allow equilibration to ambient conditions, prior to baseline thermal imaging, there is no standard temperature equilibration period length for dogs, in studies conducted on humans, frequent temperature equilibration periods of 10 min (20–22), 15 min (23, 24), 20 min (25, 26), and 30 min have been used (27). Studies on horses have reported temperature equilibration periods of 30 min or less (28–30). In a study on dogs, it was found that stable thermal imaging patterns can be found as early as 15 min after a dog's fur has been clipped (13). The dogs were allowed to assume a position of their choosing while kenneled for the 20-min equilibration period. This was done to minimize stress in the dog, however, this variation in dog position during equilibration could have caused variation in dog heat dissipation. While the dogs acted as their own controls which would minimize the effect of this variation, further research is required to determine the effect of dog position on heat dissipation is necessary.

This temperature equilibration time likely resulted in a lower post-activity skin temperature, decreasing the ΔT between the pre-exercise and post-exercise imagery. There is limited literature regarding the period of time temperature remains elevated post-activity in dogs. One study on the core body temperature of dogs after exercise found that the core body temperature remains elevated 20 min post-exercise (31). In humans it has been found that skin temperature will remain elevated 20 min post-exercise (32). Given the lack of literature, further studies to determine the optimal timing of post-exercise thermal imaging is necessary to allow for temperature equilibration without sacrificing the accuracy of the obtained ΔT.

During the thermal image capture, between the pre and post-activity imaging there was some variation in the exact positioning of the dog. A study in horses found that an increase of 1–1.5 m of distance between the subject and the thermal camera, or a change of up to a 20° difference in camera angle, had no significant effect on the temperature obtained from the captured image (33). Any small changes in dog position between the pre and post-activity images likely had a minimal effect on the findings of this study.

The comparisons between the subjects within search and treadmill groups failed to identify a significant difference in detectable thermal gradient changes between the two activities, suggesting that exercise on the treadmill can be beneficial to the training regime of dogs being trained for Search and Rescue. This similarity in the detectable thermal gradients imply similarity of muscle activity suggesting that dog involvement in a training regime which includes treadmill and rubble searches is beneficial not just in maintaining dog health but also to condition the dog to be well-adapted to the specific demands of their career in Search and Rescue. Although not documented in dogs, in people it has been found that conditioning can reduce the risk of injury (34, 35). Future research using gait analysis and activity monitors to investigate weight during locomotion and total activity may provide additional insight. Future studies isolating the treadmill and search activities are necessary to determine which activity leads to the decreased ΔT observed in this study.

## Data Availability Statement

The original contributions presented in the study are included in the article/Supplementary Material, further inquiries can be directed to the corresponding author/s.

## Ethics Statement

The animal study was reviewed and approved by University of Pennsylvania Institutional Animal Care and Use Committee. Written informed consent was obtained from the individual(s) for the publication of any potentially identifiable images or data included in this article.

## Author Contributions

CF participated in data collection, participated in study design, led the data analysis, and led the manuscript preparation. PK and DM participated in study design, data collection, and manuscript review. CO oversaw the design of the study, assisted with data collection, performed data analysis, and manuscript preparation. RR participated in image analysis and manuscript review. All authors contributed to the article and approved the submitted version.

## Conflict of Interest

RR is employed by Digatherm and provided expertise to increase the reproducibility of the data captured and image analysis but the company did not have any input in the study design or statistical analysis. The remaining authors declare that the research was conducted in the absence of any commercial or financial relationships that could be construed as a potential conflict of interest.

## Publisher's Note

All claims expressed in this article are solely those of the authors and do not necessarily represent those of their affiliated organizations, or those of the publisher, the editors and the reviewers. Any product that may be evaluated in this article, or claim that may be made by its manufacturer, is not guaranteed or endorsed by the publisher.
